# Supplementary figures and images for: BNIP3 Upregulation Characterizes Cancer Cell Subpopulation With Increased Fitness and Proliferation
Source: Front Oncol. 2022 Jul 13;12:923890. doi: 10.3389/fonc.2022.923890 (PMC9326071; doi:10.3389/fonc.2022.923890)

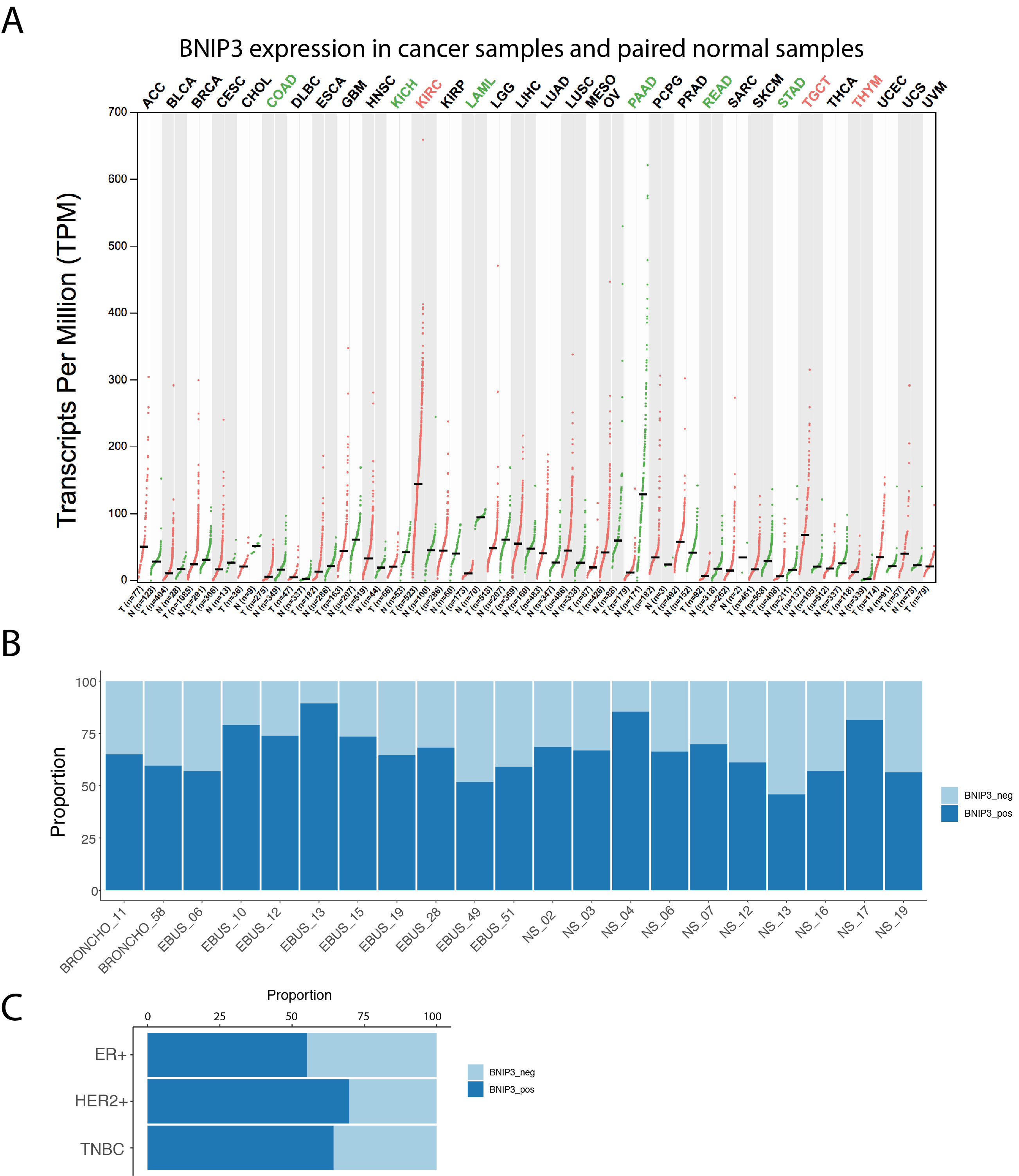

Supplement: Supplementary Figure 1 — (A). The expression of BNIP3 quantified as TPM was shown for BNIP3 in cancer samples and paired normal samples in the TCGA datasets. (B). The proportion of BNIP3 positive cancer cells in individual lung cancer sample. (C). The proportion of BNIP3 positive cancer cells for three subtypes of breast cancers. [file Image_1.jpg]
